# Biofuel production from straw hydrolysates: current achievements and perspectives

**DOI:** 10.1007/s00253-019-09863-3

**Published:** 2019-05-12

**Authors:** Volkmar Passoth, Mats Sandgren

**Affiliations:** Department of Molecular Sciences, Swedish University of Agricultural Sciences, Box 7015, SE-75007 Uppsala, Sweden

**Keywords:** Straw, Lignocellulose, Biofuels, Microbial conversion, Enzymes

## Abstract

Straw is an agricultural residue of the production of e.g. cereals, rapeseed or sunflowers. It includes dried stalks, leaves, and empty ears and corncobs, which are separated from the grains during harvest. Straw is a promising lignocellulosic feedstock with a beneficial greenhouse gas balance for the production of biofuels and chemicals. Like all lignocellulosic materials, straw is recalcitrant and requires thermochemical and enzymatic pretreatment to enable access to the three major biopolymers of straw—the polysaccharides cellulose and hemicellulose and the polyaromatic compound lignin. Straw is used for commercial ethanol and biogas production. Considerable research has also been conducted to produce biobutanol, biodiesel and biochemicals from this raw material, but more research is required to establish them on a commercial scale. The major hindrance for launching industrial biofuel and chemicals’ production from straw is the high cost necessitated by pretreatment of the material. Improvements of microbial strains, production and extraction technologies, as well as co-production of high-value compounds represent ways of establishing straw as feedstock for the production of biofuels, chemicals and food.

## Introduction

Biofuels, i.e. reduced liquid or gaseous compounds generated from renewable organic biomass, are seen as a means to decrease dependency on fossil resources, reduce greenhouse gas (GHG) emissions especially in the transportation sector and improve security of fuel supply (Passoth 2014; Valentine et al. 2012). However, biofuels are currently mainly produced from so-called first-generation substrates such as sugar cane, wheat grain or vegetable oils, i.e. resources that also can be used as human food. This use of food crops has been criticised due to potential food versus fuel competition and because land-use changes can lead to the loss of natural ecosystems (Kim and Dale 2004; Kluts et al. 2017). Therefore, substantial research has been conducted to establish biofuel production from second-generation biomass, i.e. lignocellulose such as straw or wood residues (Gnansounou 2010). Nevertheless, even lignocellulosic energy crops can compete with food production as areas are needed to produce those plants (Kluts et al. 2017). On marginal i.e. poor yielding land, the yield of dedicated energy crops is usually also poor; hence, production costs are probably high and the revenue low. There are many factors determining whether it is profitable to produce food or biofuels. If biofuel production is profitable, energy crops might be produced instead of food plants (Glithero et al. 2015; Shortall 2013; Sims et al. 2010). In contrast, cereal straw may represent an ideal resource for biofuel production, as it is a co-product of food production, and thus, its production does not compete with food generation (Townsend et al. 2017) and a high level of grain production did not have any negative effect on the utilisation of the straw as raw materials for biofuel production (Jørgensen et al. 2018).

**Fig. 1:**
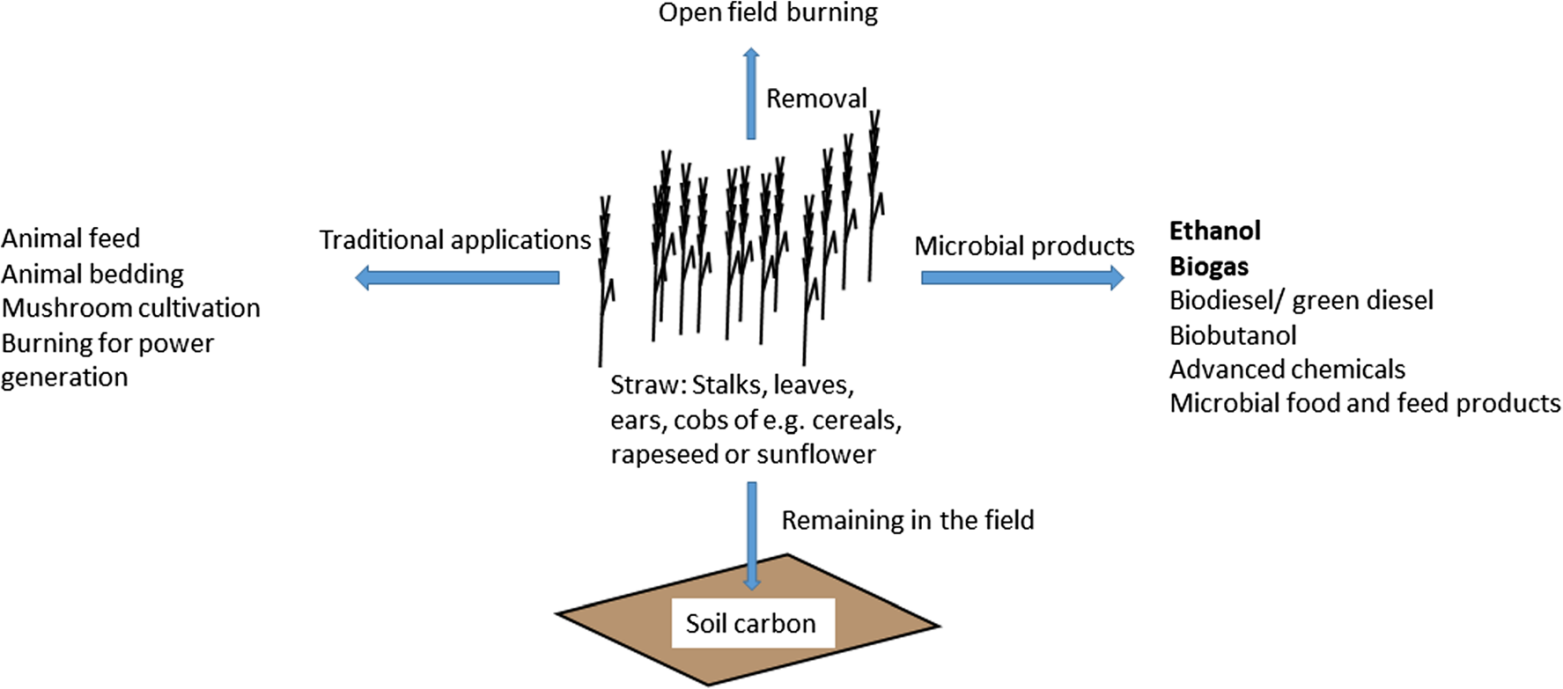
The versatile applications of straw, a side product in food production. Apart from traditional applications in agriculture, a part of it is left in the field to restore the soil carbon pool. In some areas, surplus straw is removed by open field burning, which is a waste of organic material and can substantially affect air quality in the environments of the fields. After physico-chemical and enzymatic pretreatment, microbial processes can add value to straw. There are examples of commercial production of bioethanol or biogas from straw. Research is going on to generate advanced biofuels such as biodiesel and biobutanol and chemicals from straw materials

Straw includes dried stalks, ears, cobs and leaves of e.g. cereals, rapeseed or sunflower, which are separated from the grains during harvest (Fig. 1). For instance corn stover, i.e. leaves, stalks and bare cobs from maize plants, is the most abundant straw generated in the USA (Panoutsou et al. 2017). Straw has multiple applications such as animal feed, bedding, substrate for mushroom production or power generation by burning. Nevertheless, there is a substantial surplus of straw. In several areas of the world, this surplus straw is frequently removed from the fields by open field burning. This technique, although quite convenient for the farmer, causes substantial emissions affecting the environmental balance of cereal production and the local air quality. Therefore, there are efforts to ban open field burning (Kadam et al. 2000). Biofuel production from straw can add value to this residue and reduce the consumption of fossil resources. It is not completely clear how much of the straw can be sustainably removed from fields, as there are a variety of factors impacting the amount of available straw, for instance cultivation conditions, grain cultivar, weather conditions or soil quality (Panoutsou et al. 2017; Townsend et al. 2017). For wheat straw, about 400 million tons may be globally available for biofuel production (Talebnia et al. 2010; Tishler et al. 2015). New preservation techniques for moist straw material (Passoth et al. 2013) may in some areas with high precipitation increase the amount of potentially available material (Nilsson 2000).

Straw and its conversion to biofuels and chemicals by using either chemical or bioconversion has been discussed previously (Chandel et al. 2018; Isikgor and Becer 2015; Maity 2015). This mini-review aims to provide a survey about bioconversion of straw material.

### Structure of straw and methods for pretreatment

#### Composition of different straw materials

The chemical composition and structure of lignocellulose has evolved to provide a barrier against microbial infection of the plant (Gupta et al. 2016; Tavares and Buckeridge 2015). Straw, like all lignocellulose, is a heterogenous, multicomponent material mainly built by the three major plant polymers cellulose, hemicellulose and lignin. Cellulose is a polysaccharide consisting of cellobiose subunits. Due to the β-1,4 glycosidic bounds between the glucose units building up the cellulose chain, the glucose fibrils can be very tightly packed and form crystalline structures. Hemicellulose of monocotyledons, which are the sources of straw, are branched polysaccharides built of a xylan backbone with side chains of arabinose and glucuronic acid, the latter frequently methylated. Hemicelluloses have a random and amorphous structure; they form a network in the plant cell wall, crosslinking the cellulose fibrils and lignin. Their fine structure can vary from plant to plant, tissue to tissue and even within the same molecule (Girio et al. 2010; Holtzapple 2003; Isikgor and Becer 2015; Biely et al. 2016). Lignin, in contrast to cellulose and hemicellulose, is not a polysaccharide but consists of phenylpropanoid units, which form a three-dimensional network. Lignin is hydrophobic, provides stiffness to the cell wall and resistance against insects and plant pathogens (Isikgor and Becer 2015). Some typical compositions of straw are provided in Table 1. There are, however, large variation in the proportions of the three major polymers in straw. In some studies, cellulose contents of wheat straw were found to reach almost 50% (Brandenburg et al. 2018; Saha et al. 2005). This composition variation is apparently due to differing proportions of tissues in different cultivars, i.e. the proportion of internode-, node-, leaf- and ear tissue (Collins et al. 2014).

**Table 1:** Cellulose, hemicellulose and lignin content of several straw materials (modified from Isikgor and Becer 2015)

Straw	Cellulose [%]	Hemicellulose [%]	Lignin [%]
Wheat straw	35.0–39.0	23.0–30.0	12.0–16.0
Barley straw	36.0–43.0	24.0–33.0	6.3–9.0
Rice straw	29.2–34.7	12.0–29.3	17.0–19.0
Oat straw	31.0–35.0	20.0–26.0	10.0–15.0
Corncobs	33.7–41.2	31.9–36.0	6.1–15.9
Corn stalks	35.0–39.6	16.8–35.0	7.0–18.4
Sorghum straw	32.0–35.0	24.0–27.0	15.0–21.0

Apart from organic polymers, straw also contains inorganic compounds, which after burning remain as ash. While the ash content is rather low in for instance wheat, oat or barley straw, it has a proportion of up to 20% of the total biomass in rice straw. The major element within the ash is Si. Amorphous Si polymers have been observed to form incrustations in epidermis, vascular bundle, bundle sheath and sclerenchyma tissues (Satlewal et al. 2017). The presence of silica in general had a positive correlation with the amount of cellulose, hemicellulose and lignin in the cell walls of rice plants and increases the biomass formation of rice (Zhang et al. 2015). On the other hand, it has been shown that rice plants treated with silica reduce lignin incorporation in their cell walls (Goto et al. 2003). Silica may fulfil the function of lignin as a compression-resistant compound in cell walls. Its incorporation takes less than 10% of the energy of incorporating lignin or carbohydrates into the cell wall (Satlewal et al. 2017).

### Physico-chemical and biopretreatment of straw biomass

The recalcitrance of straw necessitates pretreatment to obtain monosaccharides that can be fermented to biofuels and chemicals. Technologies for pretreating lignocellulose were developed already in the early nineteenth century, in pulp and paper production and in agriculture, to increase the digestibility of forage by ruminants. In most approaches, the material is first size reduced followed by some type of physico-chemical treatment. The pretreated lignocellulose is subsequently enzymatically saccharified (Rabemanolontsoa and Saka 2016; Jönsson and Martín 2016).

Well-established methods of physico-chemical pretreatment are concentrated or diluted acid treatment, the latter often combined with steam explosion, and alkaline treatment including ammonia-fibre expansion (AFEX). The pretreatments target different structures of the lignocellulosic material. Acid pretreatments mainly hydrolyse polysaccharides by breaking glycosidic linkages. Hydrolysis of crystalline cellulose is slower than that of amorphous hemicellulose; therefore, during acid pretreatment, hemicellulose is often degraded to sugar monomers, while cellulose is still present in polymeric form. Removal of hemicellulose is increasing the surface and opening pores for subsequent enzymatic treatment (Rabemanolontsoa and Saka 2016; Satlewal et al. 2017). Steam explosion disrupts the structure of lignocellulose due to the expansion of moisture during pressure release. Additionally, glycosidic bounds are hydrolysed due to acetic acid that is released from hemicellulose (Jacquet et al. 2012). Alkaline treatment hydrolyses linkages between polysaccharides and lignin, removes lignin and reduces crystallinity (Rabemanolontsoa and Saka 2016; Satlewal et al. 2017). All methods have advantages and disadvantages regarding the extent to which sugar monomers are released, energy demand and production of fermentation inhibitors. High silica content may cause high shearing forces, scaling or fouling of equipment (Satlewal et al. 2017). Silica had also an inhibitory effect on cellulolytic enzymes, due to non-productive adsorption of cellulase (Talukder et al. 2017). Fermentation inhibitors are formed as side products of physico-thermal pretreatment. Under acid pretreatment, sugar dehydration results in the formation of 2-furaldehyde (furfural) from pentoses and hydroxymethyl furfural (HMF) from hexoses (Brandenburg et al. 2018; Jönsson and Martín 2016). At severe pretreatment conditions, furaldehydes are further degraded to levulinic and formic acids. Moreover, acetic acid is released due to hydrolysis of the acetyl groups of hemicelluloses. A number of aromatic compounds can also be present in acid hydrolysate, most of them originating from lignin degradation (Jönsson and Martín 2016). During alkaline pretreatment, less inhibitors are generated compared to acid pretreatment. Sugars can be converted to organic acids, including saccharinic acid, lactic and formic acids and a variety of dihydroxy and dicarboxylic acids (Jönsson and Martín 2016; Satlewal et al. 2017).

It is also possible to perform biopretreatment, where the biomass is degraded with the help of microorganisms. A huge variety of both bacteria and fungi can degrade cellulose and hemicellulose to their monomers. Certain Clostridia, e.g. *Clostridium thermocellum*, have been extensively investigated for anaerobic degradation of cellulose. Actinomycetes and a variety of fungi can degrade cellulose and hemicellulose under aerobic conditions (Lynd et al. 2002). However, microbes usually consume the monosaccharides that are released during hydrolysis, and thus, sugars are typically not available for subsequent conversion to biofuels. In one study, this was overcome by co-incubation of *C. thermocellum* with a thermostable β-glucosidase. *C. thermocellum* usually takes up cellodextrins and has a rather low affinity for glucose. Due to the activity of β-glucosidase, monomeric glucose was produced from the oligosaccharides that were generated from cellulose by the clostridial cellulase activity. The combination of *C. thermocellum* and glucosidase resulted in considerable accumulation of glucose both on pure cellulose and rice straw hydrolysate (Prawitwong et al. 2013). Microbial lignin degradation is another strategy to make the polysaccharides more accessible to enzyme degradation. Delignification has been attempted using white-rot fungi such as *Trametes versicolor* or *Phanerochaete chrysosporium* or isolated lignin-degrading enzymes such as laccases. This kind of pretreatment is often time consuming when using living fungi and requires additional processes such as detoxification (Plácido and Capareda 2015). Moreover, experiments with biopretreatment require moistening of the material and in most cases start with sterilised material (e.g. Cianchetta et al. 2014), which would not be sustainable for large-scale production of biofuels and chemicals. Moist straw is quite susceptible to mould infection (Passoth et al. 2013). Straw is usually dried in the field, which implies that rainfall can affect drying and thus later utilisation of the material (Nilsson 2000). Establishing a low energy preservation system would enable even the utilisation of moist straw and thus increase the amount of raw materials accessible for straw-based production of biofuels in areas with high precipitation. Potential useful preservation systems have been developed for moist cereals, using airtight storage together with biocontrol organisms. This kind of biopreservation efficiently inhibited the growth of undesirable microbes and improved the grain characteristics for use as animal feed, such as decreased amounts of phytate (Olstorpe et al. 2010; Olstorpe and Passoth 2011). Moreover, the starch was better accessible for enzymatic degradation, resulting in improved bioethanol production from moist stored cereals (Passoth et al. 2009). The concept of airtight preservation was extended to wheat straw. Biopreservation of wheat straw with the help of appropriate biocontrol yeasts prevented mould infections, and there was even enhanced biofuel production from the biopreserved moist straw compared to dry material, indicating that biopreservation during storage can also be a part of the pretreatment (Passoth et al. 2013; Theuretzbacher et al. 2015).

### Enzymatic treatment of straw biomass

After physico-chemical pretreatment, monosaccharides are released from the polysaccharides by using enzymes. For obtaining a maximum release of sugar monomers, enzymes should be used that degrade all three major polymers: cellulose, hemicellulose and lignin (Gupta et al. 2016; Obeng et al. 2017). However, due to a lack of good lignin-degrading enzymes, commercial enzyme mixtures usually degrade cellulose and hemicellulose (Jaramillo et al. 2015). Cellulose-degrading enzymes are formed by a variety of organisms, including anaerobic and aerobic thermophilic and mesophilic bacteria, and fungi (Obeng et al. 2017). Commercial cellulolytic enzymes are usually derived from various fungal species. The most extensively studied cellulolytic enzyme systems are from the ascomycete *Trichoderma reesei* (teleomorph name *Hypocrea jecorina*) (Jaramillo et al. 2015; Obeng et al. 2017).

Traditionally, three complementary enzyme activities have been used to degrade cellulose: endoglucanases, exoglucanases and β-glucosidases (Payne et al. 2015). These enzymes are classified as belonging to the glycoside hydrolase (GH) families in the carbohydrate-active enzyme (CAZy) classification system (Lombard et al. 2014). Endoglucanases (endo-1,4-β-D-glucanases, EC 3.2.1.4) hydrolyse β-1,4 glycosidic bonds in a random manner in amorphous areas of the cellulose, generating reducing and non-reducing ends. Exoglucanases, also called cellobiohydrolases (cellulose 1,4-β-cellobiosidases) are processive enzymes (i.e. they slide along the polysaccharide chain) releasing cellobiose from either the reducing (E.C 3.2.1.176) or the non-reducing end (EC 3.2.1.91) of the cellulose molecule. β-Glucosidases (E.C. 3.2.1.21) hydrolyse cellobiose or cellooligosaccharides into glucose. Both exoglucanases and β-glucosidases are strongly inhibited by the end product of their reactions, cellobiose and glucose, respectively (Gupta et al. 2016; Obeng et al. 2017; Payne et al. 2015).

Less than 10 years ago, a new class of enzymes were discovered that act synergistically with glycoside hydrolase enzymes and play an important role in degradation of polysaccharides (Vaaje-Kolstad et al. 2010). These lytic polysaccharide monooxygenases (LPMOs) cleave glucose in a copper-mediated oxidative process at the C1 and/or the C4 position of a glucan chain (Meier et al. 2018). Activities of some LPMOs on hemicellulose have also been observed (Gupta et al. 2016). LPMOs generate chain breaks in the polysaccharide molecule, yielding additional sites for GH-enzyme activity. Due to the synergistic action of LPMOs and GHs, a lower enzyme load can be used for degrading lignocellulosic biomass, which is an important step towards economically feasible lignocellulose conversion (Obeng et al. 2017). LPMOs are classified as auxiliary activities (AA) in the CAZy classification system (Lombard et al. 2014; Meier et al. 2018).

Swollenins, also called expansins, represent an additional group of proteins involved in lignocellulose degradation. The mechanism of their activity has not yet been discovered. Nevertheless, degradation products of β-glucans have been identified after incubation with expansins, indicating activities similar to that of endo- and exoglucanases (Andberg et al. 2015).

Hemicelluloses are usually solubilised during thermal pretreatment, and some acid pretreatments obviously release sufficient amounts of sugar monomers to perform subsequent microbial cultivations on the substrate (e.g. Brandenburg et al. 2016). However, it has been pointed out that thermochemical pretreatment in many cases releases oligosaccharides, which cannot be assimilated by most of the relevant fermentation organisms. Therefore, treatment with hemicellulases has a great potential to improve the efficiency of lignocellulose-based processes (Girio et al. 2010; Biely et al. 2016). Because of the heterogeneity of hemicelluloses in different plant materials, a diverse set of hemicellulases is required, including endo-xylanases, β-xylosidases, α-glucuronidases (various types of GH families in the CAZy classification system) and acetyl xylan esterases (CE family). Alkaline pretreatment of straw yields deesterified arabinoglucuronoxylan, while non-alkaline pretreatments result in partially acetylated saccharides. Accordingly, after acid or steam pretreatment, acetylxylan esterases are required to achieve complete saccharification of straw hemicellulose (Biely et al. 2016). Oligosaccharides derived from cereal plant hemicelluloses can also be used as prebiotics (Broekaert et al. 2011). Those oligosaccharides are produced by GH11 xylanases. The shortest product of their activity consists of four xylose residues substituted with one or two arabinose residues at the penultimate xylose from the non-reducing end (Biely et al. 2016). Interactions of different hemicellulase systems with components of the cell wall have to be further elucidated to develop an optimal system for hemicellulose degradation (Biely et al. 2016; Gupta et al. 2016). After several decades of developmental work, commercial cocktails of both cellulases and hemicellulases are now available, e.g. Cellic® CTec3 from Novozyme or ACCELLERASE® TRIO™ from DuPont, and it has been demonstrated that the presence of hemicellulases also considerably improves the degradation of cellulose by cellulases (Lopes et al. 2018; Payne et al. 2015).

### Production of biofuels and other chemicals from straw

As mentioned above (Fig. 1), straw can be used as animal feed, for bedding, as substrate for mushroom cultivation or for burning (Panoutsou et al. 2017; Townsend et al. 2017). Complete removal of the straw from the field is not desirable, since this over the long term decreases the amount of soil carbon in the fields (Karlsson et al. 2017; Townsend et al. 2017). However, even after considering all these alternative utilisations, there is a surplus of straw that can be used in a biorefinery, although the annual amounts of available straw can vary, depending on for instance weather conditions and chosen cultivars (Scarlat et al. 2010; Talebnia et al. 2010). There are a variety of methods to further convert the monosaccharides derived from straw pretreatment to biofuels, including ethanol, methane (biogas), butanol or biodiesel (Chandel et al. 2018).

### Ethanol production from straw

Ethanol production is probably the most intensively investigated application of lignocellulose-derived sugars. The yeast *Saccharomyces cerevisiae*, the most established organism for ethanol production, efficiently converts glucose and other hexoses to ethanol. In addition, the yeast *Brettanomyces bruxellensis* and the bacterium *Zymomonas mobilis* can produce ethanol under industrial conditions (Blomqvist and Passoth 2015; Gupta et al. 2016); it has for instance been shown that *B. bruxellensis* can ferment oat straw hydrolysate to ethanol (Tiukova et al. 2014). Those industrial ethanol producers have a high ethanol tolerance and they can adapt to inhibitors present in lignocellulose hydrolysate (e.g. Blomqvist et al. 2011; Tiukova et al. 2014). However, these microorganisms cannot assimilate xylose and other pentoses derived from hemicellulose. To obtain an economically feasible ethanol process from lignocellulose, it is also desirable to convert the hemicellulose sugars to ethanol. Substantial efforts have been made to obtain xylose-fermenting, inhibitor-tolerant *S. cerevisiae* strains (Passoth 2014; Passoth 2017a). Finally, by a combination of metabolic and evolutionary engineering of industrial *S. cerevisiae* isolates, strains were obtained that can ferment both glucose and xylose in lignocellulose hydrolysate. These strains overexpress specific transporters for xylose, the pentose-phosphate pathway, and the xylose assimilation pathway—either xylose reductase/xylitol dehydrogenase of the xylose-fermenting yeast *Scheffersomyces stipitis* (Garcia Sanchez et al. 2010) or a codon-optimised xylose isomerase from *Clostridium phytofermentans* (Demeke et al. 2013a; Demeke et al. 2013b, Fig. 2). Second-generation ethanol production technology has in principle reached maturity for commercial production. The Danish company Inbicon established a pilot plant, which has the capacity to generate 576 kg (730 l) ethanol per hour from wheat straw (Larsen et al. 2012). The ethanol plant in Crescentino, Italy, is the world’s first second-generation ethanol plant on commercial scale. It produces about 40,000 metric tons of ethanol per year from 270,000 tons of biomass—wheat and rice straw and the energy crop giant cane (*Arundo donax*) (http://www.biochemtex.com/en/references/crescentino, accessed 1/12/2018). Construction of another ethanol plant (Clariant sunliquid) in Romania started in 2018. This plant, supported by the 7th Framework Programme of the European Union, has a projected capacity of 50,000 metric tons per year from cellulosic agricultural residues (https://www.sunliquid-project-fp7.eu/, accessed 4/4/2019). A number of commercial second-generation ethanol plants with a total capacity of about 530 million litre ethanol per year have been established in the USA and Brazil, most of them operating with straw as part of the feedstock mix. However, especially in times of low mineral oil prices, the costs for second-generation bioethanol are high compared to prices of fuels generated from fossil resources and first-generation ethanol. Due to this, competitive second-generation bioethanol production is still difficult to obtain, despite the substantial reductions in greenhouse gas emissions compared to fossil fuels. The high costs mainly arise from the need for extensive pretreatment of the lignocellulose, with concomitant high costs for equipment and thus capital (Lantz et al. 2018; Lynd et al. 2017). This might be overcome by modifying handling of feedstock. Mixing wood chips from short rotation coppice and wheat straw resulted in higher monomeric sugar release after pretreatment compared to treatment of the sole feedstocks. Blending also can mitigate supply risks due to seasonal biomass shortage. Under those conditions, prices of $60–69 per ton were calculated (Dou et al. 2019).

**Fig. 2:**
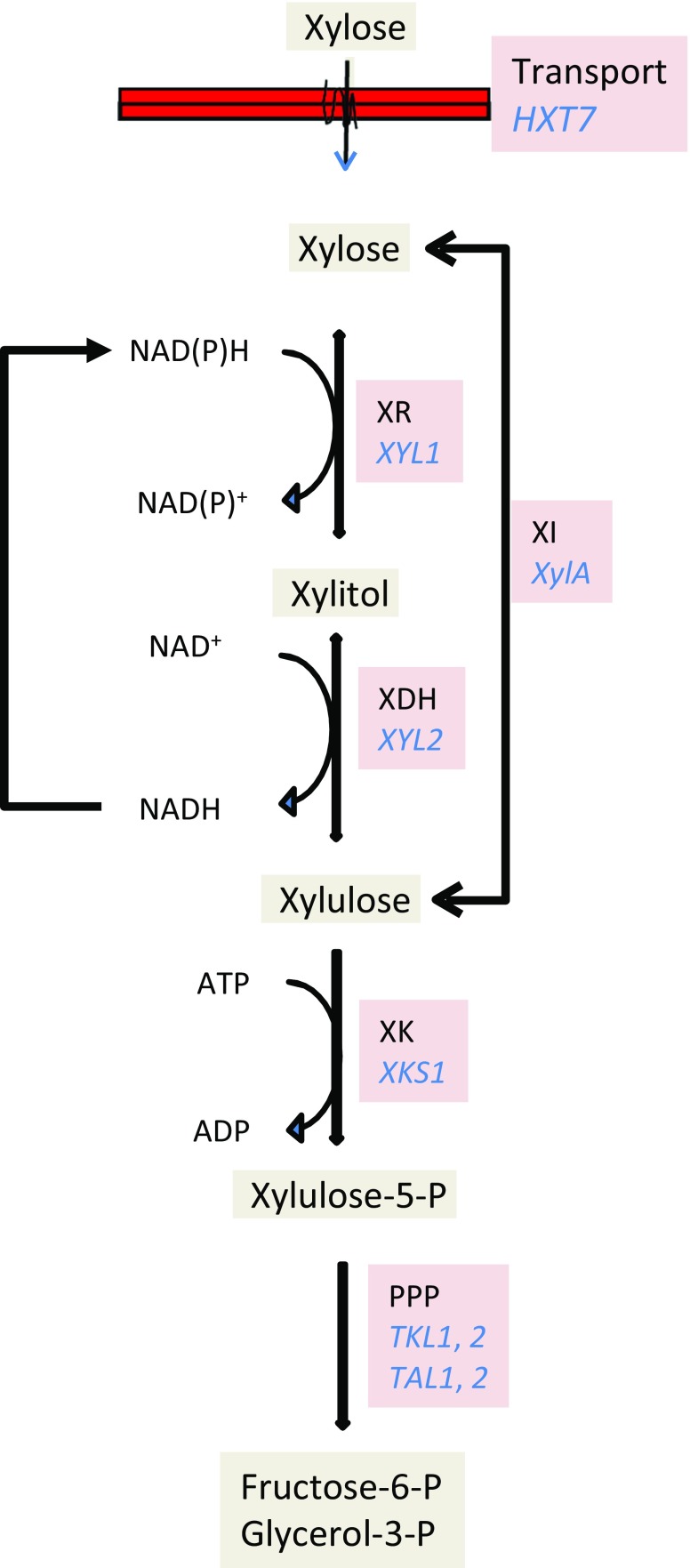
Genes overexpressed in industrial strains to obtain xylose-fermenting *Saccharomyces cerevisiae* suitable for commercial ethanol production from lignocellulose hydrolysate. The genes include the *S. cerevisiae* glucose transporter gene *HXT7*, mutated to transport both glucose and xylose, the *Scheffersomyces stipitis* genes *XYL1* encoding xylose reductase (XR) and *XYL2* encoding xylitol dehydrogenase (XDH) or a codon-optimised *XylA* from *Clostridium phytofermans* encoding xylose isomerase (XI), the *S. cerevisiae* genes *XKS1* encoding xylulo-kinase, *TKL1* and -*2* encoding transketolase, and *TAL1* and -*2* encoding transaldolase. Modified from Passoth 2017a

### Biogas from straw

Methane (biogas) production is another option when producing biofuels from straw. Biogas production by anaerobic digestion has better energy efficiency, greenhouse gas emissions and biomass conversion than ethanol production (Börjesson and Mattiasson 2008). Anaerobic digestion includes four process steps: hydrolysis of biopolymers, acidogenesis, acetogenesis and methanogenesis. During hydrolysis, carbohydrates (including celluloses and hemicelluloses from lignocellulose material), lipids and proteins are degraded into their monomers, for instance monosaccharides, amino acids and short-chain fatty acids. In acidogenesis, these compounds are further converted to organic acids (volatile fatty acids, VFAs). As side products, ammonia, carbon dioxide and other compounds are produced. In acetogenesis, VFAs are converted to acetic acid, CO_2_ and hydrogen. In methanogenesis, acetate and hydrogen are converted to methane and CO_2_. While bacterial consortia drive the first three steps, methanogenesis is performed by archea (Schnürer 2016). Since hydrolytic bacteria form part of the microbial consortium during anaerobic digestion, a pretreatment of lignocellulosic biomass is not essential for biogas production. However, due to the complexity and recalcitrance of lignocellulose, the hydrolysis step is frequently the limiting factor for methane production from lignocellulose. Therefore, a variety of physical, thermochemical or biotreatments of lignocellulose has been investigated. In principle, the same pretreatments can be used as for bioethanol production. However, losses of hemicellulose should be avoided and fermentation inhibitors may also negatively affect biogas processes. The use of sulphuric acid during pretreatment should be kept to a minimum, since sulphate-reducing bacteria may outcompete methanogens. Biopretreatments by white-rot fungi have frequently been tested, to decrease lignin content and increase availability of polysaccharides for the hydrolysis steps. Biotreatment generates less inhibitors and requires less energy than thermochemical methods; however, it is time consuming and the fungi may degrade some organic material resulting in lowered methane yields. In general, pretreatment can have positive or negative impacts on the final biogas production, and optimisation for the specific material and biogas process is required (Carrere et al. 2016; Rouches et al. 2016). There have also been attempts to combine storage of corn stover and pretreatment (Cui et al. 2012), similar to experiments with bioethanol production from wheat straw (Passoth et al. 2013). When ethanol was produced from oat straw and biogas from the residue of ethanol production, biogas production rate was considerably enhanced and the total energy output was higher than in either bioethanol or biogas production alone. This indicated that ethanol production from the initial material can serve as a pretreatment for enhanced conversion of the remainder to biogas (Dererie et al. 2011). In general, ethanol production should be connected with valorisation of residues, for instance biogas production, to obtain an energy output similar to biogas production (Lantz et al. 2018).

Lignocellulose is in general a problematic substrate for biogas production. Apart from its recalcitrant structure, it has a comparatively high C/N ratio and it has a shortage of essential trace elements such as iron, cobalt, nickel, molybdenum, selenium and tungsten. Keeping the C/N ratio between 20 and 30 is crucial for running an efficient biogas process; however, straw feedstocks have C/N ratios of 46 and 47 (oat straw and rice straw, respectively) to 60 and 63 (wheat straw and corn stover, respectively). On the other hand, low C/N ratio feedstocks such as animal waste are also problematic, as they can result in digester instability due to ammonia toxicity. Those problems can partially be overcome by running co-digestion of lignocellulose and animal waste products (Sawatdeenarunat et al. 2015, Table 2). For instance, co-digestion of rice straw and swine manure resulted in an increase in methane production by 71% (Ye et al. 2013).

**Table 2:** Anaerobic co-digestion of some straw materials (modified from Sawatdeenarunat et al. 2015)

Co-substrate	Co-substrate mixing ratio (based on volatile solids (VS))	C/N ratio	CH_4_ yield (l/kg VS)
Swine manure and rice straw	2/1	21.7	350
Chicken manure and corn stover	1/3	27.3	298
Chicken manure and corn stover	1/1.4	20	223
Chicken manure, dairy manure and wheat straw	2.7/2.7/1 (chicken manure/dairy manure/wheat straw)	25.0	235

Biogas generation is in general more sustainable and produces less emissions of greenhouse gases and health-threatening compounds than consumption of fossil fuels or open field burning of straw. However, there is still net greenhouse gas emission and global warming potential by biogas production. Under Chinese production conditions, biogas purification, biogas residue disposal and total electricity consumption are main factors to optimise for reducing negative impacts of biogas production (Wang et al. 2016).

### Butanol production

Butanol, both *n*-butanol and iso-butanol, has excellent fuel characteristics because it has higher energy density, is less corrosive and more compatible with existing engines than ethanol. Acetone-butanol-ethanol production from starchy material has been established on an industrial scale using solventogenic Clostridia (Xin et al. 2018). However, the process suffers from high costs for raw materials and too low final titres of butanol. Butanol is highly toxic to cells; thus in batch culture, the maximum butanol titres were less than 13 g/l (Mariano et al. 2011). Conversion of lignocellulosic materials to butanol has been tested, using metabolically engineered Clostridia, adding cellulolytic enzymes to fermentations or by using mixed cultures of the solventogenic Clostridia with cellulolytic bacteria. For instance, 5.5 g/l butanol could be produced from rice straw, and 10.9 g/l from corncobs. Although certain progress was made, the final butanol titres and production rates did not reach the levels necessary for commercially viable butanol production (Jiang et al. 2017; Jiang et al. 2018). Optimising lignocellulose hydrolysis and detoxification could further increase butanol production from various lignocellulose substrates; about 18 g/l were reached from barley straw, hydrolysed by dilute acid and enzyme treatment and detoxified by overliming. The fermentation strain was *Clostridium beijerinckii* P260. Optimising process parameters could further increase butanol production. Fed-batch cultivation with immobilised cells (high cell densities) and continuous removal of the butanol was most promising. A final concentration of 115 g/l butanol was reached in a fed-batch fermentation of wheat straw hydrolysate with *C. beijerinckii* P260, where butanol was continuously removed by gas stripping (Gottumukkala et al. 2017).

### Production of biodiesel and other chemicals from straw hydrolysates

After ethanol, biodiesel is currently the second most abundant biofuel in the world. Biodiesel is produced from vegetable oil, for instance rape seed, palm or soya oil. The production of vegetable oil can have a considerable greenhouse gas potential, for instance the production of one ton of palm or soya oil results in the emission of more than 2000 kg CO_2_ equivalents (Schmidt 2015). There are reports of clearing rainforest for palm oil production; therefore, movements have started in the European Union to ban the use of palm oil for biodiesel production (http://www.europarl.europa.eu/sides/getDoc.do?pubRef=-//EP//TEXT+REPORT+A8-2017-0066+0+DOC+XML+V0//EN, accessed 3/12/2018). Microbial lipids produced from lignocellulose such as straw can provide a sustainable alternative to vegetable oils. Oleaginous yeasts can accumulate more than 20% of their biomass as lipids; lipid contents of more than 70% have been reported. Lipid accumulation occurs at carbon surplus, for instance due to high C/N ratios, which are characteristic for straw hydrolysates (see above in “Biogas from straw”). At carbon surplus, the citrate cycle is inhibited in oleaginous yeasts, and citrate is transported out of the mitochondria. In the cytoplasm, citrate is converted by ATP citrate lyase to acetyl-CoA and oxaloacetate. The latter is transported back to the mitochondria, while acetyl-CoA is the basis of fatty acid synthesis, which is achieved by acetyl-CoA carboxylase and the fatty acid synthase (FAS) enzyme complex, under consumption of NADPH. Many oleaginous yeasts can convert the different hexoses and pentoses and organic acids released from lignocellulose pretreatment to lipids (Passoth 2017b; Sitepu et al. 2014). Lipid production from different lignocellulosic materials has been tested, including rice straw (final lipid concentration 11.5 g/l) (Huang et al. 2009) or corncob hydrolysate (final lipid concentration 12.3 g/l) (Gao et al. 2014). From corn stover hydrolysate, 25–30 g/l was reached (Slininger et al. 2016). Inhibitors in the hydrolysate act also against oleaginous yeasts, setting a limit for feedstock dry matter in the fermentation process, which in turn limits the amount of potential end product. Lipid-accumulating strains may be adapted to inhibitors by sophisticated feeding techniques. Brandenburg et al. (2016) discovered that the oleaginous yeast *Lipomyces starkeyi* was co-consuming acetic acid and xylose, thus increasing the pH in cultivations without pH adjustment. When the medium feed was connected to pH regulation, a self-regulating fed batch could be established, yielding the highest lipid concentration to date from the hemicellulose fraction of lignocellulose. In oleaginous *Rhodosporidium* spp. (current correct taxonomic designation *Rhodotorula*, Wang et al. 2015), fed-batch cultivation yielded the highest production levels on lignocellulose hydrolysate (Xu and Liu 2017), indicating that strains can adapt to fermentation inhibitors. However, the production price of lignocellulosic microbial biodiesel would still be too high; according to recent calculations, a minimum selling price of about $ 2.50 would be required to cover the costs (Biddy et al. 2016). On the other hand, microbial biodiesel production from lignocellulose has the potential to reach a similar energy balance as bioethanol (Karlsson et al. 2016). A number of steps in the production process of microbial biodiesel could be improved, which would significantly increase the efficiency of the whole process. This includes lipid extraction from the cells, identification of rapid lipid producers (since lipid production is an aerobic process, requiring much more energy per fermentation time compared to the in principle anaerobic ethanol production process), utilising all residues to generate co-products (such as biogas), and conversion of the crude glycerol, which is a residue of the transesterification of the microbial triglycerides to fatty acid methyl esters, to lipids (Biddy et al. 2016; Karlsson et al. 2016).

Production of high-value biodiesel and co-products will also be a means to reach competitive production prices. By fermentation with *Rhodotorula toruloides* and subsequent catalytic hydrogenation, hydrocarbons with identical characteristics to fossil diesel could be generated from corn stover (Sànchez i Nogué et al. 2018). The red colour of *Rhodotorula* species is due to the formation of carotenoids, mainly β-carotene. Carotenes are widely used as colourants and antioxidants in the food, feed, pharmaceutical and cosmetic industries. Co-production with lipids can improve the economic viability of biodiesel production (Schneider et al. 2013). Furfural is another high-value compound that can be produced from wheat straw. This dehydration product of pentoses is one major platform chemical to produce biofuels, fuel additives and other compounds. It is a side product of thermochemical pretreatment and acts as an inhibitor in the fermentation broth. Currently, there is no technology for synthetic furfural production; it has to be produced from lignocellulose. Current technologies for furfural production are not very efficient and they damage the cellulose, so that its glucose monomers cannot be converted to biofuels (Machado et al. 2016). At Latvian State Institute of Wood Chemistry, a novel technique for furfural extraction has been developed, allowing an efficient extraction of furfural from lignocellulosic material without extensively damaging the cellulose fraction. Recently, it has been shown that it is possible to co-produce furfural and ethanol or lipids (from 1 kg straw, 110 g furfural—69% of the theoretical maximum—and 111 g ethanol or 33 g lipids were produced). Pentoses were in principle completely converted to furfural, and the hydrolysate contained the easily fermentable glucose. Moreover, the hydrolysate had a low content of fermentation inhibitors (Brandenburg et al. 2018). It is also possible to utilise the lipids of oleaginous yeasts for other purposes, for instance as ingredient in fish feed, to replace vegetable oil such as palm oil. As it is not necessary to extract the oil or to run transesterification, this approach can also be a valuable step towards a sustainable economy, taking into account the environmental impact of palm oil production (see above) (Blomqvist et al. 2018).

Polyhydroxybutyrate (PHB) is a polyhydroxyalkanoate (PHA), which is produced by certain bacteria as intracellular storage compound. PHAs can be used as bioplastics, replacing fossil-based plastics produced from mineral oil components. Production of PHAs by microorganisms has been investigated during the last years; however, production costs were usually too high to achieve a substantial replacement of plastic from fossil resources. Identification of cheap carbon sources for the microbial production of PHA may be one approach to decrease production costs. AFEX-pretreated wheat straw was used for PHB production with *Burkholderia sacchari*. An intracellular PHB concentration of 72% was achieved within 61 h of cultivation, corresponding to 105 g/l PHB (Cesário et al. 2014). Rice straw hydrolysate obtained from alkaline pretreatment was used as substrate for *Ralstonia eutropha* to produce PHB. An intracellular PHB concentration of 75% was achieved within 48 h of cultivation, corresponding to a total PHB concentration of 11.4 g/l (Saratale and Oh 2015).

1,4-Butandiol is another large-volume chemical that is currently mainly produced from fossil resources. It has a global market of about two million tons per year and has a range of applications as platform chemical for the production of plastics and other products. *Escherichia coli* has been engineered to produce 1,4-butandiol from a variety of sugars with comparatively high yield and productivity in a commercial scale (Burgard et al. 2016). LCA showed a positive effect compared to production from fossil resources. However, also negative effects were observed, including terrestrial acidification and marine eutrophication because of ammonia fertilisation during production of biomass (Forte et al. 2016). The impact of biomass production on the environmental sustainability needs to be regarded to exploit the full potential of utilising straw as raw material for the production of biofuels and biochemicals.

## Conclusion and outlook

Straw is a lignocellulosic agricultural residue with great potential as feedstock in biotechnological production processes (Fig. 1). As it is co-produced with cereal grain and other food raw materials, its production does not compete with food production and will not result in land-use changes. Substantial research towards converting this feedstock to biofuels and other high-value compounds has been conducted during the last years. There is a long and growing list of interesting chemicals that can be generated from straw materials (Chandel et al. 2018; Gupta et al. 2016). Given the high dependency of the global economy on fossil resources, there is an urgent need to find alternative ways of producing fuels, chemicals and food. Moreover, producing high-value compounds from agricultural residues will add value to the agricultural industry, increasing the economic attractiveness of green technologies. Establishing technologies to produce different fuels and chemicals from straw will create opportunities for generating products best adapted to the local conditions and market demands. Frequently, production of more than one biofuel can be combined, such as ethanol and biogas or biodiesel and biogas (Karlsson et al. 2016; Lantz et al. 2018). The whole chemical complexity of the biomass should be used, if possible. Lignin is an underutilised resource, but there are ongoing efforts to identify enzymes to degrade and utilise this biopolymer as well (Gupta et al. 2016). On the other hand, lignin is currently often burned to provide process energy. It can also be transferred back to the soil to maintain the soil organic carbon content and, in the long term, to prevent greenhouse gas emissions (Karlsson et al. 2016; Karlsson et al. 2017; Lantz et al. 2018). For any application, a careful analysis of both environmental and economic consequences has to be performed to obtain a sustainable replacement for fossil resources.
